# Superhelical Destabilization in Regulatory Regions of Stress Response Genes 

**DOI:** 10.1371/journal.pcbi.0040017

**Published:** 2008-01-18

**Authors:** Huiquan Wang, Craig J Benham

**Affiliations:** UC Davis Genome Center, University of California Davis, Davis, California, United States of America; Helmholtz Centre for Infection Research, Germany

## Abstract

Stress-induced DNA duplex destabilization (SIDD) analysis exploits the known structural and energetic properties of DNA to predict sites that are susceptible to strand separation under negative superhelical stress. When this approach was used to calculate the SIDD profile of the entire Escherichia coli K12 genome, it was found that strongly destabilized sites occur preferentially in intergenic regions that are either known or inferred to contain promoters, but rarely occur in coding regions. Here, we investigate whether the genes grouped in different functional categories have characteristic SIDD properties in their upstream flanks. We report that strong SIDD sites in the E. coli K12 genome are statistically significantly overrepresented in the upstream regions of genes encoding transcriptional regulators. In particular, the upstream regions of genes that directly respond to physiological and environmental stimuli are more destabilized than are those regions of genes that are not involved in these responses. Moreover, if a pathway is controlled by a transcriptional regulator whose gene has a destabilized 5′ flank, then the genes (operons) in that pathway also usually contain strongly destabilized SIDD sites in their 5′ flanks. We observe this statistically significant association of SIDD sites with upstream regions of genes functioning in transcription in 38 of 43 genomes of free-living bacteria, but in only four of 18 genomes of endosymbionts or obligate parasitic bacteria. These results suggest that strong SIDD sites 5′ to participating genes may be involved in transcriptional responses to environmental changes, which are known to transiently alter superhelicity. We propose that these SIDD sites are active and necessary participants in superhelically mediated regulatory mechanisms governing changes in the global pattern of gene expression in prokaryotes in response to physiological or environmental changes.

## Author Summary

DNA in vivo experiences regulated amounts of untwisting stress. If sufficiently large, these stresses can destabilize the double helix at specific locations. These sites then become favored locations for strand separations. Gene expression and DNA replication, the two major jobs of DNA, both require the strands of the duplex to be separated. Thus, events that affect the ease of strand separation can regulate the initiation of these processes. Stress-induced DNA duplex destabilization (SIDD) has been implicated in mechanisms regulating several biological processes, including the initiation of gene expression and replication. We have developed computational methods that accurately predict the locations and extents of destabilization within genomic DNA sequences that occur in response to specified stress levels. Here, we report that the easily destabilized sites we find in the *Escherichia coli K12* genome are statistically significantly overrepresented in the upstream regions of genes encoding proteins that regulate transcription. In particular, the regions upstream of genes that directly respond to physiological and environmental stimuli are more destabilized than are those regions of genes that are not involved in these responses. These results suggest that strong SIDD sites upstream of participating genes may be involved in transcriptional responses to environmental changes.

## Introduction

Genomic DNA not only encodes proteins and RNAs, but also plays active roles in mechanisms regulating many biological processes, primarily through interactions with proteins and other molecules. Although strictly lexical analysis of genomic DNA sequences has been a focus of contemporary biology, it is becoming increasingly clear that the physical, chemical, and structural properties of the DNA molecule can play key roles in the onset or progression of many regulatory events. Some of these attributes are not directly related to sequence in any simple way.

Because initiation of either transcription or replication requires transient separation of the strands of the DNA duplex, mechanisms regulating either process must closely control where and when this strand opening occurs. Any event that alters the local stability of the DNA duplex at such regulatory regions has the potential to affect the ease of initiation of gene expression or DNA replication. Even stability changes of only a few kilocalories, which are far too small to open the duplex directly, can have a profound influence on the equilibrium of an opening reaction that is mediated by other molecules [1]. In particular, the untwisting stresses resulting from negative DNA superhelicity can drastically destabilize specific genomic regions [2,3]. If strand opening at a regulatory site is the rate-limiting step in initiating a process, then this superhelical destabilization can have a major effect on the frequency with which that process occurs. In this way, changes in the in vivo level of superhelicity could strongly affect specific regulatory events.

Stress-induced DNA duplex destabilization (SIDD) is a complex process in which the behavior of each base pair affects that of every other base pair experiencing the stress. Because of this interactive character, the sites susceptible to SIDD cannot be identified strictly by their local sequence attributes. Whether a specific site opens under stress depends on its context as well as its sequence, and may vary in complex ways with stress level [3].

In vivo, DNA superhelicity is tightly controlled by a variety of processes. In prokaryotes, a basal level of superhelicity is imposed, primarily by the activities of topoisomerase enzymes. Because gyrases are ATPases, this level can vary between growth and stationary phases according to the energy charge of the cell [4]. Transient changes of superhelicity also occur in response to a variety of environmental factors, including osmotic stress, anaerobic stress, and temperature shock [5–10]. Translocation of RNA polymerase produces a bow wave of overtwist (positive superhelicity), and a wake of undertwist (negative superhelicity) [11]. Further, the effective boundaries of superhelical domains may vary with protein binding events. Therefore, the level of negative superhelicity experienced by a promoter can vary according to the transcriptional activities and orientations of neighboring genes, with the binding of specific proteins, and during the accommodation to environmental or physiological changes. Transcription of one gene may affect the expression of neighboring genes through transcription-induced changes of superhelicity. In particular, the destabilization experienced in an intergenic region separating divergently oriented genes can increase drastically when either or both genes are actively being transcribed [12]. Negative superhelicity can be sequestered by binding of architectural molecules, such as histones in eukaryotes or HU proteins in bacteria. Hence, altering the pattern of architectural binding also can affect the amounts or distributions of superhelical stresses in neighboring regions.

Changing the level of superhelicity imposed on a region of DNA can have a variety of effects on the expression of the genes it encodes. Both in vivo and in vitro experiments have shown that the activities of some promoters are enhanced by negative superhelicity, while others are inhibited and still others are unaffected [13,14]. A variety of DNA structural properties may account for some of these differential effects. For example, supercoiling may affect transcription by stabilizing DNA looping and/or DNA bending; by promoting the formation of non-B form structures such as cruciforms, quadriplexes, or Z-DNA; or by altering the stability of the duplex at specific sites [15].

One particularly intriguing example of the latter mechanism is the *ilv*P_G_ promoter of *Escherichia coli.* This promoter is upregulated approximately 5-fold by binding of integration host factor (IHF) to an upstream regulatory region when the DNA is negatively supercoiled, but when it is relaxed, IHF binding has no effect [16]. The mechanism of this regulation involves superhelically driven DNA denaturation. In the absence of IHF, negative superhelicity destabilizes the IHF binding site, which is approximately 100 bp upstream from the −10 region of the promoter. Because IHF binds to duplex DNA, its binding forces this region back into a double helix, and removes the bound site from the competition for stress-driven opening [17]. Under these circumstances, the imposed negative superhelicity causes the next-easiest site to open, which is in the −10 region of the promoter. Thus, this mechanism of IHF-mediated transcriptional regulation involves a binding-induced transmission of destabilization from the IHF binding site into the promoter [18].

This example illustrates one mechanism by which superhelical stresses can transduce the influence of protein binding to remote positions, which is an essential component of many regulatory processes. It also shows that regulatory duplex destabilization need not occur at the site where opening is required by the regulatory process. Opening at other sites, as mediated or transduced by protein binding, can be essential components of regulatory mechanisms.

In prokaryotic cells, the expression levels of many genes undergo dramatic changes when conditions that affect superhelicity are altered, for example in transitions between growth phase and stationary phase. The resulting differences in the patterns of global gene expression have been extensively documented [8–10]. An accumulation of evidence has led to the suggestion that modulation of DNA superhelicity in prokaryotes may serve as a regulator of gene expression in vivo, both locally and on a global, genome-wide scale [4,19]. Although superhelicity may affect regulatory processes in many different ways, it is clear that stress-induced duplex destabilization is a central component of several mechanisms [4]. Even moderate levels of destabilization can dramatically affect the rate of initiation of any process in which strand separation is required.

We have developed computational methods to calculate the SIDD properties of any DNA sequence under superhelical stress [20–23]. These methods evaluate the statistical–mechanical equilibrium distribution of a population of identical molecules under the given conditions. From this distribution, two properties are calculated that together depict the state of stability of each base pair. These are the probability *p*(*x*) of the base pair at position *x* being open, and the incremental free energy *G*(*x*) of the set of states in which that base pair is always open [21]. These two parameters are inversely related: pairs that have a high probability of opening have a small SIDD energy *G*(*x*). These calculations have no free parameters; all conformational and energy parameters are given their experimentally measured values. However, they predict locations and extents of superhelically driven strand opening to high accuracy in all molecules where the appropriate experiments have been performed [3,17,21,24–26]. This gives confidence in the accuracy of results when these methods are applied to other sequences, on which experiments have not been performed.

Sites of predicted superhelically driven duplex destabilization (SIDD sites) have been shown not to occur at random, but instead to be closely associated with specific types of regulatory regions. Examples include replication origins in yeast, bacteria, and viruses [1,27,28], and regions regulating transcription of specific genes from a variety of organisms [3,17,24,28,29]. Certain noncoding regions containing promoters or terminators are highly destabilized, while transcribed regions remain stably duplexed under the stresses imposed by negative superhelicity. Results from several experiments have implicated SIDD in the mechanisms regulating a variety of biological processes, including transcription initiation [12,29,30], replication [31,32], and chromosomal scaffold/matrix attachment [25,33].

We previously reported the complete SIDD profile of the E. coli K12 genome [28], which was calculated using a new algorithm specific for long genomic DNA sequences [22]. Our results indicate that strong SIDD sites are relatively rare: at a physiologically reasonable superhelical density of *σ* = −0.06, less than 1% of the E. coli genome has *G*(*x*) < 0 kcal/mol. Also, the locations of destabilized sites show an intriguing pattern: the DNA duplex within coding regions is mostly highly stable, while the predicted sites with strong SIDD propensities are statistically enriched in intergenic regions separating divergently and tandemly oriented genes, but not in intergenic regions separating convergently oriented genes. For example, the strongest SIDD sites, those whose minimum *G*(*x*) values satisfy *G*_min_ < 0 kcal/mol, cluster at divergent intergenic regions at frequencies that are more than 27 standard deviations higher than what would be expected if these SIDD sites were randomly distributed along the genome. Strong destabilization occurs at tandem intergenic regions at frequencies more than 19 standard deviations higher than the expected random frequency. This pattern appears to be universal throughout the prokaryotic domain; it has been found in every one of the more than 200 prokaryotic genome sequences we have analyzed to date [34]. We have exploited this result to develop a SIDD-based promoter prediction strategy for prokaryotes [35]. This approach finds fewer than half of the promoters because not all promoters are destabilized by stresses. However, its predictions have such low false positive rates that the sites it identifies have high probabilities of being correct.

Because strong SIDD sites are found in some putative promoter-containing regions but not in others, it is of interest to investigate whether specific families of genes share common SIDD properties in their upstream flanks. In this report, we document a statistically significant enrichment of genes whose 5′ flanks contain strong SIDD sites within specific families of functionally related genes. In particular, the strongest SIDD sites in the E. coli K12 genome are associated with genes encoding transcriptional regulators. We also examine the SIDD properties of functional families within the genomes of numerous other free-living bacteria as well as several obligate parasitic bacteria and endosymbionts. We show that the upstream regions of genes whose protein products function in transcription have a highly significant association with the strongest SIDD sites in the free-living bacteria, but not in the endosymbionts and obligate parasitic bacteria. We also find that genes in E. coli K12 that directly respond to physiological and environmental stimuli are more likely to have highly destabilized 5′ flanks than do others, even within the regulator family. Finally, we show that the downstream genes in a regulatory pathway commonly have the same SIDD properties in their 5′ flanks as the controlling transcriptional regulator gene itself. In light of these results, we suggest that strong SIDD sites are active and necessary participants in the regulatory mechanisms governing changes in the global pattern of gene expression in response to physiological or environmental changes in prokaryotes.

## Results

### Distinct Classes of Genes in the E. coli K12 Genome Have Statistically Significantly Different SIDD Properties in Their Immediate 5′ Upstream Regions

To analyze how the SIDD sites occurring immediately upstream from the genes in the E. coli K12 genome vary among gene/product types, we used the GenProtEC classification scheme [36]. GenProtEC partitions these genes into 13 disjoint sets—12 functional product types, plus genes of unknown function. These classifications are based on experimental evidence where available, and on sequence similarity otherwise. A total of 4,518 genes have been classified this way in the May 2006 version of the GenProtEC database used in this study.

Next, we categorized these genes according to the amount of destabilization found in their 5′ upstream regions. For this purpose, we used the SIDD profile of the complete E. coli K12 genome, calculated at linking difference *σ* = −0.06 as described in the Methods section. We considered three definitions of what constitutes an upstream region. To examine only promoter destabilization, we first limited consideration to the region within 50 bp upstream of the start of the open reading frame (ORF) of each gene. However, because the 5′ ORF boundary often does not coincide with the transcription start site, in some cases the promoter may not in fact be included in this 50-bp window. In other cases (such as the IHF-mediated activation of the *ilv*P_G_ promoter described above), a regulatory SIDD site is located further upstream, although the promoter it regulates is within the 50-bp window. For both of these reasons, we next widened this window to encompass the 250 bp immediately upstream from the 5′ end of each ORF. Because prokaryotic genomes have a gene-dense character, this window is likely to contain whatever promoter a gene may have as well as other regulatory sites. In both of these cases, windows of the given length were selected without regard for whether this interval was entirely intergenic or overlapped another gene, and regardless of gene type (RNA or protein coding) or position within an operon. As a third case, we also examined the full 5′ intergenic region upstream from each gene, regardless of its length, provided there was one. It was found that 3,656 genes had annotated upstream intergenic regions, with 3,024 distinct intergenic regions located directly upstream from one or more genes. Of these, 632 are bounded by divergently oriented ORFs (each of these ORFs is counted separately in the gene count, while the region itself only counts once in the enumeration of intergenic regions), and 2,392 are bounded by tandemly oriented genes. In what follows, we use the 250-bp definition of upstream regions, unless otherwise noted. Results using the other definitions are similar, and are provided in the supplementary materials (Datasets S1 and S2).

We next grouped the genes according to the extent of destabilization in their upstream regions. We partitioned the genes into nine disjoint categories, called SIDD0 through SIDD7 and SIDD8+, according to the minimum value *G*_min_ of the destabilization energy *G*(*x*) in any SIDD site that overlapped their upstream regions. Briefly, a gene is placed in category SIDD*i, i* = 0,...,7, if it has *G*_min_ < *i* and it is not in SIDD(i − 1). The SIDD8+ class contains those genes whose upstream regions do not overlap SIDD sites that are destabilized below *G*(*x*) = 7 kcal/mol. In particular, they have *G*(*x*) > 7 kcal/mol throughout their upstream regions. (The procedure used to make this classification is fully described in the Methods section.) Because smaller values of *G*(*x*) correspond to sites that are more easily destabilized by superhelical stresses, this arrangement organizes the genes according to the ease of opening of their upstream regions, the SIDD0 genes being most easily destabilized. We performed this partitioning separately for each of the three definitions of upstream regions described above.

**Figure 1:**
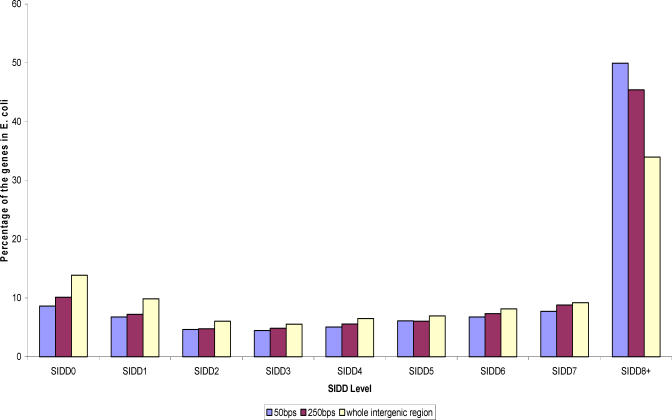
Genes in the E. coli K12 Genome according to Their Upstream SIDD Sites The genes in the E. coli K12 genome are grouped according to the most destabilized SIDD sites in their whole upstream regions (yellow columns), or the most destabilized SIDD site in their immediate 50-bp or 250-bp upstream regions (blue or red columns), respectively. The former classification shows the highest SIDD0 fraction because there are fewer of them; 3,656 genes have 5′ intergenic flanks, while all 4,518 genes in the May 2006 release of GenProtEC are included in the other cases.

The distributions of E. coli K12 genes into SIDD groups are summarized in Figure 1 for each of the three definitions of what constitutes an upstream region. As shown there, the largest SIDD category in all cases is the SIDD8+ group; for example, 45.4% of all E. coli genes are not destabilized in their immediate 250-bp upstream regions. In all cases, however, the next largest category is SIDD0, which are the most strongly destabilized sites. This holds despite the fact that these are the most rare SIDD sites in the genome [28]. Although there are only 506 SIDD0 sites in the entire genome, 457 of them occur in upstream flanks of genes.

Each SIDD group contains genes from every functional category. To assess whether the genes in our SIDD groups are associated with specific functional categories in a statistically significant way, we needed to determine how they would distribute at random. As described in the Methods section below, if the genes in the SIDD*j* group were randomly distributed between those in functional category *k* and those not in this category, the result would follow a hypergeometric distribution [37]. This allows us to calculate the probability of attaining at random within a given functional category at least the observed number of genes within a specified SIDD group (see the Methods section below for a full description). To determine which of these associations are statistically significant, we calculated the log odds probability of the genes in each SIDD group being distributed as observed in each functional category. Cases whose probabilities are sufficiently small are correspondingly unlikely to have occurred through a random process. (Small probabilities correspond to large log odds scores. Here, natural logarithms are used, so a random probability of *p* < 0.05 corresponds to a log odds score greater than 2.997.)

**Figure 2:**
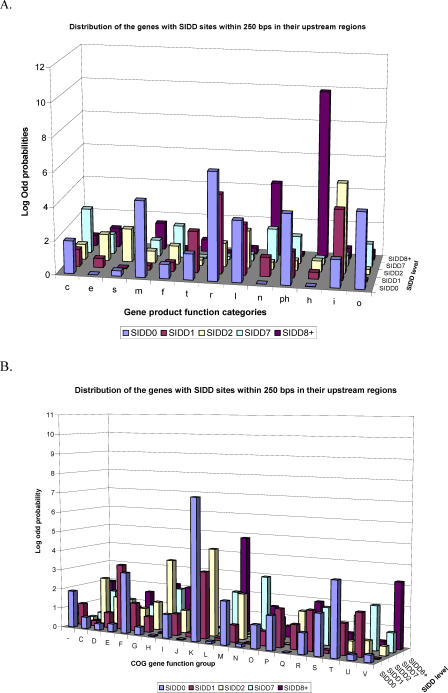
The Statistical Significances of the Associations of Genes in Functional Categories with the Extent of Destabilization in Their Upstream Flanks (A) GenProtEC functional categories; (B) COG classifications. In both cases, the immediate upstream 250-bp regions are used. Figures for the other two definitions of 5′ flanks are similar, and are given in Datasets S1 and S2.

Figure 2A depicts the log odds scores for the distributions of genes in the SIDD0, SIDD1, SIDD2, SIDD7, and SIDD8+ groups across the 13 GenProtEC functional categories. Here, we show the results for upstream regions of 250 bp in length. (The corresponding graphs for upstream intergenic regions and for 50-bp upstream regions are similar to that shown here, and are presented in Dataset S1.) One sees that genes in five functional categories (*m, r, l, ph,* and *o*) are statistically significantly associated with SIDD0 sites at the *p* < 0.05 level. Category *l* contains only 12 genes, too few for this association to be regarded as meaningful. The categories *ph* (phenotype) and *o* (other—unknown functions) are catch-alls, each containing genes with many different types of functions. This leaves the two large and specific categories of regulators (*r*) and membrane proteins (*m*), the upstream flanks of whose genes are statistically significantly enriched in the strongest SIDD sites. Of these, the highest statistical significance occurs in the category of genes that encode regulators. There, a significance of *p* < 0.01 occurs in both the SIDD0 and SIDD1 groups. On the other hand, categories of RNA genes (*n*), and genes of external origin (*h*) have statistically significantly elevated numbers of genes in the SIDD8+ group, corresponding to stable 5′ upstream regions. These conclusions remain essentially unchanged for each of the three ways of defining upstream regions. (The only substantive difference is that genes encoding enzymes [*e*] are highly significantly represented in the SIDD8+ category when one uses intergenic regions, but not when either 50-bp or 250-bp upstream regions are used. The difference may be due to the frequency with which these genes occur in operons.)

We note that genes internal to operons may not have promoters in their 5′ flanks. And, in the annotation of the E. coli K12 genome used here, 862 genes either overlap or directly abut their upstream neighbor, and hence do not have flanking 5′ intergenic regions at all. For these reasons, each of our three ways of identifying 5′ upstream regions will include many genes whose 5′ flanks do not contain promoters. The fact that we find statistically significant associations of SIDD sites with the 5′ flanks of specific gene families despite this dilution by nonfunctional regions speaks to the strengths of these associations.

Although GenProtEC provides the most experimentally based classification of genes into families, it is only available for *E coli* K12. To treat other organisms, as is done below, we use the functional categories defined by the Clusters of Orthologous Groups (COG), which classifies the protein products of genes into 21 groups according to their functions [38]. We analyze the patterns of SIDD site association with genes whose products are in the various functional COG groups using the same approach as was used for GenProtEC. The results for E. coli K12 are shown in Figure 2B (and in Dataset S2). A similar pattern of enrichment for strong SIDD sites is found using the COG classifications as was shown above for GenProtEC. The upstream regions of the E. coli K12 genes in the “Transcription” category (COG category K) are statistically most significantly associated with strong SIDD sites, attaining levels of *p* < 0.001 for the SIDD0 group and *p* < 0.05 for both the SIDD1 and SIDD2 groups. The two functional COG categories of signal transduction mechanisms (category T) and nucleotide transport and metabolism (category F) also are enriched in strong upstream SIDD sites at the *p* < 0.05 level.

Because the GenProtEC and COG classification systems form distinct groupings of genes, it is not straightforward to compare the patterns of distribution of upstream SIDD sites found for each system. The statistical significance of associations between SIDD sites and genes in different functional categories may depend on the specific ways each classification is made. We thus compared the genes in those categories of the GenProtEC or COG classifications that showed significant association with the strongest SIDD sites in E. coli K12 genome. We found that 68 out of 83 genes in the nucleotide transport and metabolism COG group F (about 82%) are contained in the much larger enzymes GenProtEC group (*e*). Similarly, the GenProtEC membrane protein group *m* is widely split among the COG groups, with 29% of its genes in the not in COG (−) group, 16% in the function unknown (S) group, 12% in the general function prediction (R) group, 15% in the cell wall/membrane biogenesis (M) group, 10% in the cell mobility (N) group, and 10% in the intracellular trafficking and secretion (U) group, respectively. Thus, the statistical significance observed above for the nucleotide transport and metabolism (F) group by COG and the membrane proteins (*m*) group by GenProtEC are likely tied to the specific criteria used in making these classifications.

### Strong SIDD Sites in the E. coli K12 Genome Are Statistically Significantly Associated with Genes Functioning in Transcription

Both the GenProtEC and COG classifications show that genes functioning in transcription in E. coli K12 are statistically significantly associated with strong upstream SIDD sites. As described above, in the COG classification system, the highest statistical significance of this association is achieved by the transcription group (K), while in the GenProtEC classification, it is attained by the regulator (*r*) group.

**Figure 3:**
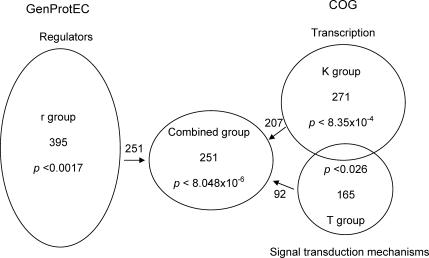
Gene Sets Functioning in Transcriptions Assigned by GenProtEC and COG The sizes of each set are indicated. The construction of the combined set is shown. (We note that the COG categories are not disjoint, so the same protein can appear in more than one COG group.) The statistical significances of the association of each set with SIDD0 sites are also given.

Figure 3 shows a comparison of the compositions of these groups. The GenProtEC *r* group contains 395 genes, 62% of which fall into either COG group K (transcription) or group T (signal transduction mechanisms). Specifically, 207 of the 271 genes in COG group K (75%) and 92 of the 165 genes in COG group T (56%) are contained in the GenProtEC regulator group, *r.* (One important difference between the GenProtEC and the COG classifications is that in the former the groups are disjoint, whereas in the latter they may overlap; COG can assign a protein to more than one functional category.)

We developed a combined group containing all genes of the COG T and K groups that are also members of the GenProtEC *r* group. As shown in Figure 3, this combined group comprises a set of 251 genes. We have assessed the statistical significance of the occurrence of SIDD0 sites in the upstream regions of the genes in this set to be *p* < 8.05× 10^−6^. This is the highest statistical significance of any association examined here. This set includes transcriptional activators, repressors, and two component systems. Unlike enzymes such as DNA helicases or RNA polymerases, the activities and expression levels of the genes for these proteins are actively modulated under different environmental or physiological conditions.

### The Upstream Regions of E. coli K12 Genes that Respond Directly to Physiological and Environmental Stimuli Are More Destabilized Than Are Those of Genes that Do Not So Respond

Much work has been done to identify those genes that respond to environmental or physiological changes, and the pathways by which these responses occur. A particular focus has been on determining the “first responder” genes, which initiate and orchestrate adaptive responses. To illuminate the possible roles of strong SIDD sites in these responses, we have compared the occurrence of such sites in the 5′ flanks of specific E. coli K12 genes that respond to different environmental stresses. We first examined genes that have been shown experimentally to be critical “first responders,” mediating the adaptation to the environmental stress.

The protein RpoS, also known as σ32, plays a pivotal role in most environmental or physiological stress responses [39]. A SIDD0 site was found to be located at the 5′ upstream region of the *rpoS* gene. In contrast, only a SIDD7 site is found in the 5′ upstream region of the gene encoding its close relative, the “housekeeping” σ70 factor RpoD. Gyrase, comprised of the GyrA and GyrB subunits, is the main enzyme in E. coli that introduces negative supercoils into its genomic DNA, while topoisomerase I is the predominant enzyme that relaxes negative supercoils [40]. The *gyrA* and *gyrB* genes have SIDD sites at levels 2 and 5, respectively, in their 5′ upstream regions, while the *topA* and *topB* genes have upstream SIDD sites at levels 6 and 8, respectively. The histone-like proteins HU (encoded by the *hupA* and *hupB* genes) and HNS are involved in the global control of DNA supercoiling during stresses [41,42]. The 5′ upstream regions of their encoding genes are highly susceptible to destabilization, with SIDD sites at level 0 for *hupA* and *hupB,* and at level 1 for the gene encoding HNS. In contrast, IHF (encoded by *himA* and *himD*) is an abundant DNA binding protein that may not be involved in supercoiling control during stresses. Its genes have relatively stable 5′ upstream regions (SIDD4 for *himA* and SIDD6 for *himD*). The transcriptional regulators CRP and FNR are key global regulators in a variety of environmental stress conditions [39,43], while LacI regulates the expression of the *lac* operon in the presence or absence of latose. The genes encoding these proteins have substantially different SIDD properties; a SIDD1 and a SIDD0 site are located at the 5′ upstream regions of the *crp* and *fnr* genes, respectively, while the 5′ upstream region of lacI has only a relatively stable SIDD7 site. Outer membrane porin (Omp) family proteins play critical roles in osmotic stress [44], as do the transport proteins encoded by the proV operon [45]. The upstream regions of these two genes are more destabilized than those of the *tyrP* and *fadL* genes. The *tyrP* gene encodes a tyrosine-specific transporter; and the protein encoded by the *fadL* gene is a membrane-bound long chain fatty acid transporter. The expressions of *tyrP* and *fadL* genes are regulated by the availability of their exogenous substrates (i.e., tyrosine and long chain fatty acids, respectively [46,47]). The SIDD properties of the upstream flanks of these genes are summarized in Table 1. Taken together, these results indicate that the upstream regions of genes which are known to respond to environmental or physiological stresses tend to be highly destabilized, while those of other genes that do not so respond tend not to be strongly destabilized.

**Table 1:**
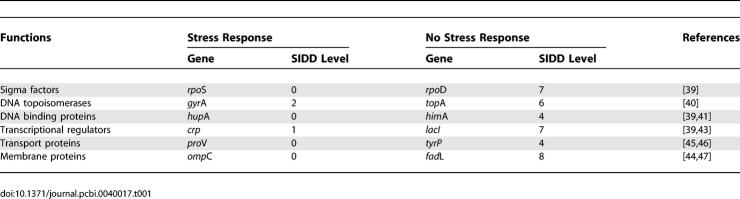
SIDD Properties in the Upstream Regions of Genes Responding to Physiological and Environmental Stresses Are Contrasted with Those of Functionally Matched Genes That Do Not Respond

### Strong SIDD Sites in Free-Living Bacteria, but Not in Endosymbionts or Obligate Parasitic Bacteria, Are Statistically Significantly Associated with the Upstream Regions of Genes in the Transcription COG Group

The statistically significant association of the strongest SIDD sites with upstream regions of those E. coli K12 genes whose products function in transcriptional control suggests that superhelical duplex destabilization at these sites may play roles in the mechanisms regulating their expression. The fact that many of these SIDD-associated genes function in adapting the organism to changing environmental or physiological conditions suggests that superhelically modulated destabilization may be involved in mediating this adaptive transcriptional response. To explore this question further, we performed the same analysis as was described above to assess the associations of upstream SIDD sites with the COG transcription group K for 18 sequenced strains of obligate parasitic bacteria and endosymbionts and for 43 strains of free-living bacteria. The rationale for this approach is that free-living bacteria must adapt their metabolisms and their gene expression patterns to changing environmental conditions, whereas obligate parasitic bacteria and endosymbionts, because they live in stable environments, need not do so. So, if upstream SIDD sites function in mechanisms of adaptation to environmental change, they would be expected to be more prevalent in free-living bacteria than in endosymbionts.

This analysis found strong SIDD sites (either SIDD0, SIDD1, or SIDD2) to be associated with upstream regions of genes in COG group K at the *p* < 0.05 level in only four of the 18 analyzed genomes of obligate parasitic bacteria and endosymbionts. These are two of the seven analyzed strains of *Chlamydia/Chlamydophila* and two of 11 strains of *Mycoplasma.* (The accession numbers of all analyzed strains and the data for each on the significance of these associations are given in Dataset S3.) In most of these obligate parasitic bacteria and endosymbiont strains, the most significant associations of strong upstream SIDD sites with COG groups occurs in the “not in COG” or “function unknown” categories. The result is markedly different for free-living bacteria. In almost all the analyzed genomes from free-living organisms, we find that strong SIDD sites are associated with upstream regions of genes in the transcription COG group K at the *p* < 0.05 level. This result was found for all 14 analyzed strains of *E. coli, Salmonella,* and *Shigella,* and for 24 of the 29 analyzed strains of *Bacillus, Staphylococcus,* and *Streptococcus.* Thus, strong upstream SIDD sites are preferentially associated with genes functioning in transcriptional regulation in free-living bacteria that must adapt to different environments, but not in obligate parasitic bacteria or endosymbionts, which only experience a single environment.

This difference may result from the reductive evolution undergone by endosymbionts [48]. In adapting to their specialized intracellular niches, these organisms over time have lost many of the genes that encode the transcriptional regulators that are needed to orchestrate adaptations to different environments. Free-living opportunistic pathogens, in contrast, must cope with much more volatile and variable environments. Thus, their genomes must encode a number of transcriptional regulators that enable them to rapidly adapt to major environmental changes. Both the presence of significant associations of strong SIDD sites with genes functioning in transcription in free-living bacteria and the absence of these associations in most obligate parasitic bacteria and endosymbionts support the notion that these strong SIDD sites may play active roles in regulating the expression of their associated genes. These results lead us to speculate that some of these strong SIDD sites may be regulatory elements in transcriptional mechanisms that enable bacteria to respond to environmental or cellular physiological changes.

### If a Pathway Is Controlled by a Transcriptional Regulator Whose Gene Has a Highly Destabilized Upstream Region, then the 5′ Flanks of the Genes in the Pathway it Controls Usually Also Contain Strong SIDD Sites

We next examined the genes encoding transcriptional regulatory proteins to determine whether specific families are enriched for strongly destabilized 5′ upstream regions. Using the methods employed above, we found two outstanding subgroups of genes whose upstream regions are enriched in SIDD0 sites at the *p* < 0.01 level. These are genes that encode transcriptional regulators in the LysR family (also called LTTR, the family of LysR-type transcriptional regulators), and genes for two-component signal transduction systems. These are the two largest families of positive transcriptional regulators in prokaryotes [49–51].

The proteins in each of these families regulate gene expression in response to changes of environmental and nutritional conditions. The activities of LysR regulators are usually induced by small molecules such as secondary metabolites or ions, while two-component systems consist of a sensor protein and a cognate response regulator. When the sensor protein detects signals indicating specific environmental changes, it phosphorylates its cognate regulator, which in turn regulates a cascade of changes in the transcription of specific genes.

#### SIDD in the 5′ flanks of genes in the LysR family.

Of the 46 transcriptional regulators in the LysR family of E. coli K12, 31 are direct neighbors of their (putative) target genes, while the other 15 regulate a variety of targets that are dispersed in the genome. In all 31 cases of direct neighbors, the two genes are divergently oriented, sharing a common regulatory region. Because both genes have the same region as intergenic 5′ flank, they clearly are destabilized there to the same extent.

**Figure 4:**
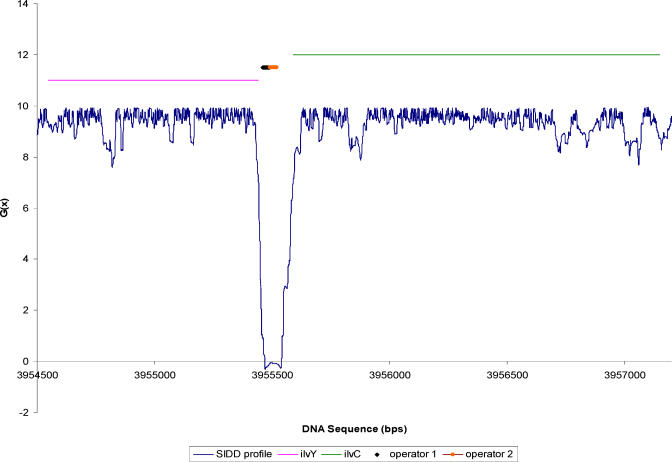
SIDD Profile of the Genomic Region Containing the Gene for the LysR-Type Transcription Regulator ilvY and its Target Gene *ilvC* These form a divergently oriented gene pair that are known to be coordinately regulated by transcriptional coupling [53].

The divergent arrangement of these genes allows simultaneous bidirectional control of both, possibly involving transcriptional coupling between them through transcriptionally induced superhelicity. An example where this is known to occur is the *ilv*YC operon. Here, IlvY is in the LTTR family, and *ilvC* is the target gene it regulates. The SIDD profile for this operon is given in Figure 4. It has been demonstrated that transcriptional coupling of these two genes is mediated by the negative supercoiling generated by RNA polymerase translocation [52,53].

**Figure 5:**
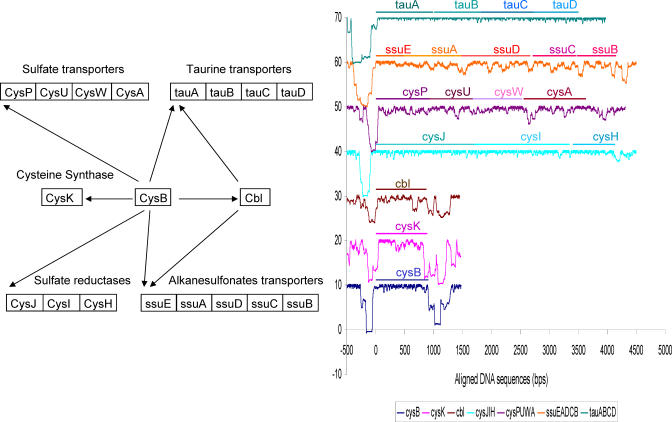
SIDD Profiles of the Genomic Regions Containing Genes (Operons) in the CysB Regulatory Pathway The left panel shows a diagram for CysB regulatory pathway, according to the information provided in [54]. The right panel shows the SIDD profiles for the operons encoding the genes for the proteins in the CysB regulatory pathway. These profiles are aligned at their translation start sites.

We next considered whether the other 15 LTTR genes and their targets also have similar SIDD properties at their 5′ upstream regions. We found that in the eight cases where the LTTR genes have the most highly destabilized upstream regions, the genes they regulate (where known) also have highly destabilized 5′ flanks. An example is provided by the *cysB* regulatory pathway, which positively controls cysteine biosynthesis in E. coli K12 [54]. The *cysB* gene whose product regulates this system is not linked to any of its many target genes, a group that includes the operons *cysJIH, cysPTWA,* and *cysK.* CysB positively regulates the expressions of these operons, and also directly regulates the expression of the *tauABCD* and *ssuEADCB* operons together with another LysR transcriptional regulator Cbl, whose gene is also controlled by CysB. Strikingly, every one of these operons, and the *cysB* gene itself, all have highly destabilized upstream regions. This is shown in Figure 5, which displays the SIDD profiles of these operons and their upstream regions aligned at their translation start sites.

#### SIDD in the 5′ flanks of genes in two-component systems.

Two-component systems are the predominant signal transduction strategy used by bacteria to respond to a variety of environmental stresses [50,51]. There are 27 known two-component systems in *E. coli,* each consisting of a sensory kinase and a response regulator. In 21 of these, the kinase and regulator genes of the system are tandemly oriented neighbors, and are transcribed together within a single transcriptional unit (operon). The primary promoter governing operon expression occurs in the 5′ upstream region of the first gene of the operon.

**Figure 6:**
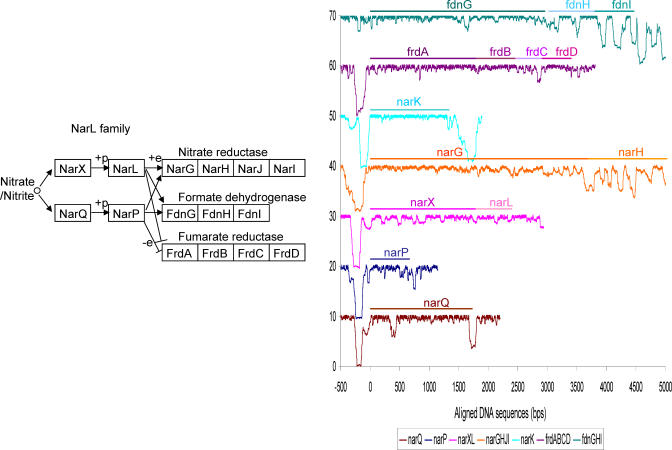
SIDD Profiles of the Genomic Regions Containing Genes (Openons) in Nitrogen Metabolism Regulatory Pathway The left panel shows a diagram for nitrogen metabolism regulatory pathway, redrawn with permission from [56]. The right panel shows the SIDD profiles for the operons encoding the genes for the proteins in the nitrogen metabolism regulatory pathway. These profiles are aligned at their translation start sites.

As described above, the upstream flanks of the first genes in these operons are statistically significantly enriched in strongest SIDD (i.e., SIDD0) sites at the *p* < 0.01 level. In five of the six cases where the two genes comprising a system are separated, both genes in the pair are destabilized in their 5′ flanks to similar extents. Two representative examples are NarP/NarQ and NarL/NarX. These systems regulate anaerobic gene expression when nitrate and/or nitrite are available [55]. The *narX* and *narL* genes form an operon *narXL,* while the *narP* and *narQ* genes are separated by about 300 kbp in the genome. There are SIDD0 sites located in the upstream regulatory regions of both the *narQ* and *narP* genes, as well as in that of the *narX* gene, the first gene in the *narXL* operon. These systems are known to regulate four transcriptional units, the *narK* gene and the *narGHJI, fdnGHI,* and *frdABCD* operons [56]. The SIDD profiles of these genes and operons are shown in Figure 6. The upstream regions of six of these seven transcriptional units are strongly destabilized, the exception being *fdnGHI.* In contrast, the upstream regions of internal genes in all these operons are substantially more stable.

In summary, we find that the upstream regulatory regions of genes in the same regulatory pathway commonly have similar SIDD properties to those of the gene for the transcriptional regulator that controls the pathway. Examples include *cysB, narXL,* and *narP/Q,* all of which have highly destabilized upstream regions. The target genes of these transcriptional regulators are not limited to the GenProtEC regulators category. Indeed, most of these genes are in the enzymes, transporters, membrane protein, or other functional categories. The observation that there exist highly destabilized sites in the upstream regulatory regions of almost all of the genes (operons) in the same pathway suggests that strong SIDD sites may be functionally related or a common component of the mechanisms governing the expression of these genes.

### SIDD Properties Do Not Correlate with Microarray Experiments

Microarrays have been used to identify sets of E. coli genes whose RNA abundances change in response to a variety of specific environmental stresses. Here, we consider experiments addressing three cases—the stringent response, the response to osmotic shock, and the response to an inhibitor-induced relaxation of DNA supercoiling [8,9,57]. We have examined whether the distribution of genes among SIDD groups is significantly different for those genes determined by these studies to respond to the given stress than it is for the genome as a whole. We found that the SIDD distributions of upregulated and downregulated genes do not differ greatly from that of the complete gene set. (These distributions are presented in Dataset S4.) We performed a χ^2^ goodness-of-fit test to assess the statistical significance of whatever differences there might be, where the null hypothesis was that the proportions of genes in the various SIDD groups were the same for the entire genome as they were for a given set of genes that were seen in an expression profile experiment to respond to the stress. In no case could the null hypothesis be rejected at the *p* < 0.05 level. This shows that the association of SIDD sites with upstream gene flanks in the overall E. coli genome is similar to the pattern of association for those “stress response” and “supercoiling response” gene groups that were identified by these microarray experiments.

We also examined the responses in these microarray experiments of the 12 genes shown in Table 1, whose roles in various stress responses have been individually determined by careful experimentation. As shown in the table, six of these genes are known to play roles in stress responses, and the other six matched genes are known not to be involved in stress responses. In none of the examined microarray experiments were these sets discriminated. This suggests the possibility that the microarray experiments do not accurately resolve responding genes from nonresponding genes in these cases.

However, there are two other reasons for the above-noted lack of correlation between the microarray results and the SIDD properties within 5′ flanks of genes. First, microarray experiments do not differentiate between genes that are directly affected by the stress, and genes that are indirectly affected by it through its influence on other factors within highly complex regulatory networks. It is possible that many, perhaps even most, genes noted in a microarray experiment to change their RNA abundances in response to the altered condition do so through indirect effects. If some, but not all, of the direct effects involved SIDD-based mechanisms, then the proportion of responding genes with the appropriate SIDD properties may not be large.

Second, because SIDD sites are differentiated from non-SIDD sites only by their behavior under negative superhelicity, it is tempting to assume that mechanisms of transcriptional regulation involving these sites would induce superhelical activation. However, an examination of the known mechanisms shows that this is not correct. In some cases, SIDD-based mechanisms upregulate their genes in response to negative superhelicity, and in other cases they down-regulate them. A SIDD-based regulatory mechanism would require a specified level of superhelicity to act, but once that level is attained, the mode of regulation that occurs could be either to upregulate superhelically activated genes or to downregulate superhelically inhibited genes. In fact, these two categories of genes, as discriminated in the microarray experiment, do not have significantly different distributions of SIDD properties. Therefore, the expectation that SIDD sites would be found primarily in the 5′ flanks of genes that are upregulated by superhelicity is unlikely to be correct.

## Discussion

In previous work, we have shown strong SIDD sites to be statistically significantly associated with those intergenic regions in the E. coli K12 genome that contain promoters [28,34]. However, although SIDD sites are concentrated at promoters, not all promoters have SIDD sites [35]. In this study, we examined the identities of those genes that have strong SIDD sites in their 5′ flanks.

We first considered genes clustered into functional categories, as assigned by either the GenProtEC or the COG classification schemes. We found that strong SIDD sites in the E. coli K12 genome are statistically significantly associated with the upstream regions of genes whose products function in transcription. These genes include transcriptional activators, repressors, and two-component systems that play important regulatory functions in gene expression. In this analysis, we considered all genes within a category, regardless of their orientation or position within putative operons. The observed significance would presumably have been enhanced had we limited consideration to the first genes within transcriptional units. This was not done here because in our view, this subset could not be identified with sufficient reliability.

Next, we examined the association of strong SIDD sites with the upstream regions of those genes in other organisms whose products function in transcription. For this purpose, the COG classification was used because GenProtEC is specifically limited to *E. coli.* We found that in 38 of the 43 genomes of free-living bacteria that were examined, strong SIDD sites are significantly enriched in the 5′ flanks of genes whose products are involved in transcription. However, a similar enrichment was noted in only four of 18 genomes of obligate parasitic bacteria or endosymbionts. This suggests that strong SIDD sites may play roles in the mechanisms by which the transcriptional program of an organism adapts to environmental or physiological changes. Because obligate parasitic bacteria or endosymbionts do not experience such changes, they are not expected to have retained these adaptive mechanisms.

We compared the SIDD properties of the 5′ flanks of specific genes across functional categories. We found that those genes that reportedly respond to environmental or physiological stresses tend to have more highly destabilized upstream regions. In addition, if the gene for a primary transcriptional regulator has a strongly destabilized 5′ flank, then the control regions of the genes that are in the pathways it regulates are usually similarly highly destabilized, regardless of the functional category in which they occurred.

These results, taken together with those from our previous studies, suggest that strong SIDD sites may serve as functional elements in specific transcriptional regulatory mechanisms. This is known to be the case in specific examples. The *ilv*P_G_ promoter of E. coli K12 is upregulated by IHF binding when the DNA is negatively supercoiled, but when it is relaxed, IHF binding has no effect [16]. The mechanism of this regulation involves the binding-induced transmission of superhelically driven DNA denaturation from the binding site to the −10 region of the promoter [17]. The *leuV* operon is regulated by a similar mechanism, with Fis binding mediating transmission [4,58]. In this case, the binding can be either activating or inhibiting, depending on whether the transmitted destabilization goes. The strongly destabilized SIDD site in the yeast CUP1 promoter was observed to be open under superhelical stress, which was minimal condition for in vitro transcriptional initiation from this promoter [29]. In humans, the activation or repression of the *c-myc* gene is governed in part by the binding of the bi-functional FBP protein to a single stranded region [30]. Whether this binding is activating or inhibiting depends on the extent of opening of this region, which is affected by transcriptionally driven superhelicity.

In all of these examples, the observed regulatory strand separations are determined by the level of superhelicity of the DNA involved. It is important to note, however, that SIDD site destabilization by superhelicity can serve in mechanisms either of activation or of inhibition. In light of the associations documented here, we posit that changes in DNA superhelicity also may dictate the regulatory functions of the strong SIDD sites 5′ to genes whose products are involved in adapting to changing conditions.

The DNA of most prokaryotes is negatively supercoiled in vivo. During environmental stresses or cellular physiological changes, such as heat or cold shock or transitions from growth phase to stationary phase, the level of DNA superhelicity has been observed to transiently change before the cells become fully adapted to their new condition [6]. Such changes in DNA supercoiling have been proposed to be global regulators of gene expression [4]. The results from this study specifically suggest that strong SIDD sites, which are the most susceptible sites in the genome to stress-induced destabilization, may be important components of the mechanisms by which changes in DNA supercoiling exert the regulatory effects that alter transcription. Several lines of evidence support this suggestion.

We have shown here that strong SIDD sites are statistically significantly associated with upstream regions of genes encoding transcriptional regulators. In general, the genes within an organism are organized into extremely complex, multileveled transcriptional regulatory networks, with the transcriptional regulators as “hubs” [59]. Genes whose expression levels are tuned to superhelicity can function as “first responders” to those environmental changes that affect supercoiling. The prevalence of SIDD sites in the 5′ flanks of genes encoding transcriptional regulators suggests that the first response to environmental changes may involve regulatory superhelical strand separations in their promoters. Thus, DNA supercoiling would both directly regulate the expression of these “first responder” genes, and indirectly regulate expression of the downstream genes in the pathways that are controlled by these regulators. The propagation of these regulatory effects through the network would change the global pattern of gene expression, adapting the cell to the new environmental or physiological conditions.

We also have documented a significant association of strong SIDD sites in the 5′ flanks of genes functioning in transcription in free-living bacteria, but not in obligate parasitic bacteria and endosymbionts. This supports the suggestion that strong SIDD sites may play important roles in mediating the transcriptional responses to environmental changes. Obligate parasitic bacteria and endosymbionts live in relatively stable environments, while free-living bacteria must adapt to changing conditions. During the course of their evolution from a free-living to an intracellular lifestyle, endosymbionts have lost many of the genes that encode transcriptional regulators. We speculate that when they lose these “environmental responsive” genes, their associated, putatively regulatory, strong SIDD sites were lost as well.

We have found that the upstream regions of genes that reportedly respond to environmental stresses are more easily destabilized on average than are those of other genes within the same functional category. However, in the living cells, genes in different functional categories are interconnected through transcriptional regulatory pathways or networks. In addition to genes in the “regulator” functional category, the members of a pathway may include the genes in “enzyme,” “transporter,” “membrane protein,” or other categories. The observation that there exist highly destabilized sites in the upstream regulatory regions of almost all of the genes (operons) in specific pathways suggests that strong SIDD sites may serve as a “signature” element for these pathways, a common motif that may serve a common regulatory function. For bacteria to be viable under all kinds of environmental conditions, some genes must actively respond to environmental or physiological stresses, either by up- or by down-regulation, while others need to stably express to maintain essential life activities with minimal fluctuation. We speculate that strong SIDD sites are the “signature” regulatory elements for those genes that actively respond to the changes of DNA supercoiling during environmental changes. This suggests that strong SIDD sites may be important regulatory elements in prokaryotes that participate in mechanisms for modulating global gene expression in responses to physiological and environmental changes. Their activities may be mediated either directly or through interactions with other events such as protein binding, as is the case of the *ilv*P_G_ promoter, and their effects may be either activating or inhibitory.

**Figure 7:**
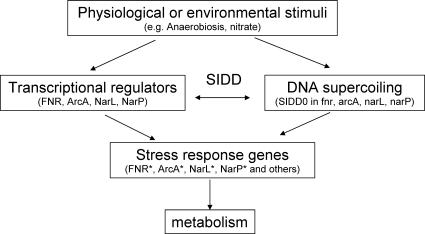
Proposed Model of SIDD-Mediated Transcriptional Regulation Strong SIDD sites are proposed to be important regulatory elements for global gene expression in response to environmental or physiological stresses. doi: 0.1371/journal.pcbi.0040017.g007

Figure 7 presents a diagram of a model for the cascade of events consequent on a physiological or environmental change. When a bacterium experiences such a change, its transcriptional program must be modified in response. Two interacting components of the regulatory machinery are considered here—transcriptional regulators and DNA supercoiling. The changed levels of DNA supercoiling that result from an environmental or physiological transition, acting in concert with protein binding events, either activate or repress the expressions of specific sets of genes which are involved in adapting to these changes. In some mechanisms, these components may influence the response to environmental or physiological changes through their effects on the duplex stability at strong SIDD sites. This is possible because changes in DNA supercoiling alter the torsional stresses that drive DNA duplex destabilization, which will be concentrated at the strong SIDD sites. Acting in concert with transcriptional regulators, strong SIDD sites may serve as switches to tune the expression of certain sets of genes, operons, or pathways. In this way, changes in the activities of global regulators, and particularly the “first responders,” together with supercoiling-induced duplex destabilization events, provide a large number of possible mechanisms by which a bacterium may adjust its physiological activities (metabolism) to adapt to changes in its living environment or physiological state.

We consider a specific example of global gene regulation in *E. coli K12* during an aerobic-to-anaerobic transition according to our proposed model. As the oxygen concentration decreases, the global regulators FNR and ArcA are activated. Activation of FNR occurs through its direct sensing of oxygen, while activation of ArcA occurs by phosphorylation by ArcB kinase [10,60]. When either nitrate or nitrite is available as an anaerobic electron acceptor, NarL and NarP are also activated by their corresponding sensor kinases NarX and NarQ [55]. Simultaneously, the negative DNA supercoiling of the genome is transiently partially relaxed in response to anaerobiosis. Because the upstream regulatory regions of the *fnr, arcA, narP,* and *narXL* genes all have strong SIDD sites, these changes of superhelicity may modulate their levels of transcription through mechanisms involving duplex destabilization. The interaction of FNR, ArcA, NarL, and NarP with their own structurally altered control regions may further down- or upregulate their own expression levels, as well as the expression of the other genes they regulate. In this way, the overall activities of these global regulators, and the abundances of the products of the genes they regulate, are altered to suit the anaerobic conditions. A new state of metabolism is ultimately achieved that attunes cellular physiological activities to the absence of oxygen.

The above example is based on results from this and our previous studies. Although the model it illustrates is relatively simplistic, it provides a possible explanation of how changes in DNA supercoiling may serve as a global transcriptional regulator in response to environmental or physiological stresses. Specifically, changes of superhelical stress affect transcriptional events through their influence on the extent of DNA duplex destabilization at strong SIDD sites. If these sites coincide with protein binding sites, then binding of this protein can further affect the distribution of destabilization. In practice, transcriptional regulation commonly involves interactions among proteins, and between proteins and DNA secondary structures. However, the precise mechanisms by which strong SIDD sites may function in these regulatory mechanisms may well be highly complex. For example, in the CysB regulatory pathway shown in Figure 5 above, the strong SIDD sites in the 5′ flanks of the genes involved each has a specific location relative to its transcription start site, a different degree of destabilization in response to superhelicity, and its own position relative to whatever protein binding sites are present near that specific gene. The specific roles of destabilization events in the regulation of this pathway may be complicated by these interacting factors.

## Methods

### Calculation of SIDD profiles.

The SIDD properties of all complete genomes treated here were analyzed using a previously described method whereby the DNA sequence is partitioned into overlapping windows and each window is analyzed separately [22]. Each window has length *N* = 5,000 bp, with successive windows offset by 500 bp. For circular chromosomes, each base pair appears in 10 windows. However, for linear chromosomes, the last window may not be exactly 5,000 bp long, and the most end-proximal 500 bps occur in a single window. The final values of the probability *p*(*x*) and the destabilization energy *G*(*x*) for the base pair at position *x* are calculated as the weighted averages of their computed values in each of the windows that contain that base pair. A detailed description of this algorithm has been presented elsewhere [22].

In these calculations, all conformational and free energy parameters are given their experimentally measured values, so there are no free parameters [3,21]. We assume superhelix density *σ* = −0.06, a moderate physiological value [61]. This analysis of the complete E. coli K12 genome required approximately 4 h to execute on a 28 node Apple X-Serve cluster, each node containing dual 1 GHz G4 processors.

### Gene classifications.

There are several ways that the genes in the E. coli K12 genome can be classified into disjoint functional categories according to their gene types and/or the cellular roles of their products. In this study, we used the GenProtEC classification scheme (http://genprotec.mbl.edu) [36]. GenProtEC is based on the wealth of experimental information available for *E. coli,* but for that reason is limited to this organism. For organisms about which less information is available, alternative classifications must be used, such as the COG system [38]. Because the COG system is computationally based, inferring orthologous function based on sequence homology, it is less reliable but can be applied to any prokaryotic genome. Therefore, it may be used to place in functional categories the genes of organisms other than E. coli K12*,* and to make comparisons between prokaryotic genomes. We note that GenProtEC partitions the genes, so each gene appears in exactly one category, while COG allows a gene to be placed in more than one category.

In this work, we have considered both the GenProtEC and the COG classifications of the genes in E. coli K12. For other microorganisms, we used COG classifications. In principle, the analyses performed in this paper can be applied to any other gene classification scheme.

### SIDD sites and SIDD groups.

A SIDD site is defined as a maximal consecutive set of base pairs which all have *G*(*x*) ≤ 8.0 kcal/mol. The SIDD sites in a genome are partitioned into eight disjoint groups, according to the minimum value *G_m_* of *G*(*x*) they attain. For example, the SIDD0 group consists of those SIDD sites whose minimum *G*(*x*) values satisfy *G_m_* ≤ 0.0 kcal/mol; SIDD1 consists of those sites with 0.0 < *G_m_* ≤ 1.0 kcal/mol; and so on up through SIDD7. For those sequences in which the *G*(*x*) of each base pair is greater than 7 kcal/mol, and hence which are not significantly destabilized, an additional group SIDD8+ is designated.

### Classification of genes by their 5′ SIDD properties.

Previous work has shown that the E. coli K12 genome is strongly destabilized in those intergenic regions that either are known or inferred to contain promoters [28]. Here, we examine whether this destabilization is associated in statistically significant ways with genes in specific functional categories. For this purpose, the 5′ upstream regions of genes must be classified according to their SIDD properties. We do this in three ways.

Because open complex formation is required at the −10 region of a promoter, we first consider the SIDD properties of the interval within 50 bps upstream (i.e., in the 5′ flank) of each ORF. This is done for every gene, without regard for whether the entire interval involved is intergenic, or whether all or part of it falls within the coding region of a neighboring ORF. However, in some cases a promoter lies more than 50 bp upstream from the start site of its ORF. In other cases (such as the IHF-mediated activation of the *ilv*P_G_ promoter described above), a regulatory SIDD site is located further upstream, although the promoter it regulates is within the 50-bp window. For both of these reasons, we next widened this window to encompass the 250 bp immediately upstream from the 5′ end of each ORF. This window is likely to contain whatever promoter a gene may have, as well as other regulatory sites and possibly coding sequences from its neighbor gene. (Because coding sequences have been shown to have very few SIDD sites, their presence in these regions is not expected to affect the results of our analyses.)

Because of the operon structure of prokaryotic genomes, these approaches include 5′ regions of genes that do not have their own promoters. This will dilute the results so that any statistical significance found is likely to be understated. However, this approach applies to every ORF in the genome.

Alternatively, we consider the SIDD properties of the entire intergenic region that is upstream from an ORF, regardless of its length, provided there is one. This approach does not include the 862 E. coli genes that either directly abut or overlap their 5′ neighbors. Also, cases are known where regulatory regions controlling a specific gene overlap or are contained within the coding region of its neighbor. These cases also are not considered in this approach.

Each of these three strategies determines a collection of upstream 5′ flanks of genes. For each such region we find all SIDD sites that overlap it, if any. We then determine the SIDD category of each overlapping SIDD site. In cases where there are multiple such sites, we choose the most strongly destabilized category. In this way, we place each included gene into a category determined by the SIDD group of the most destabilized SIDD site (if any) occurring within the upstream region that abuts it. We place a gene in category SIDD*j* if its 5′ flank is overlapped by a SIDD*j* site, and if this is the most destabilized site that overlaps it. We note that the term “SIDD*j*” now refers to two distinct groupings—it is a classification of SIDD sites and a classification of genes according to the extent of destabilization in their 5′ flanks. However, the context in which the term is used will make clear which meaning is intended.

### Test for the association of SIDD sites with specific gene classifications.

As described above, we have two ways of classifying genes into functional categories (GenProtEC and COG), and three ways of examining the SIDD properties of upstream regions. Thus, there are six different ways of examining how SIDD properties are associated with upstream regions of those genes within specific functional categories. We perform a similar analysis on each case. However, the genes considered must both be in a functional category and have an upstream region of the type being considered. We denote by *N* the number of genes for which both types of information is available, noting that in some cases *N* will equal the number of ORFs in the genome, while in other cases it will be a smaller number.

There are two pieces of information associated with each gene—its functional category and the SIDD group SIDD*j* of the most destabilized site that intersects its upstream region. To assess whether the SIDD groups are associated with specific functional categories in a statistically significant way, we need to determine how they would be distributed at random. Suppose that *φ_k_* is the number of genes in functional category *k*, and that *σ_j_* is the number of genes in the genome whose upstream regions intersect SIDD*j* sites, *j* = 0,…,8+. Let *s_jk_* be the number of these genes that also are in functional category *k*. If 5′ flank destabilization occurred at random with respect to functional categories, then the probability of finding exactly *s_jk_* SIDD*j* genes among the *φ_k_* genes in category *k* would follow a hypergeometric distribution [37]:

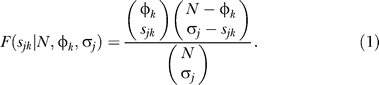



So, the probability *p*(*n*) of there being at random at least *n* genes in this category whose upstream regions are in SIDD*j* is:

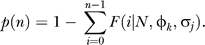



From these formulas, one may directly compute the probability that there are at least *y* genes in this category whose upstream regions are destabilized below *m*, if desired.

Suppose there are *n_jk_* SIDD*j* genes in functional category *k*. If the probability *p*(*n_jk_*) of there being at least that many genes of this type is less than a specified cutoff (usually either 0.05 or 0.01), we conclude that the association of SIDD*j* sites with that functional category is correspondingly unlikely to arise by chance, and hence is statistically significant. To better display these associations, we compute

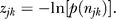
This transformation gives larger values to more improbable states. For example, a state where *p*(*n*) = 0.05 has *z* = 2.996, while one whose probability is *p*(*n*) = 0.01 has *z* = 4.605.

We perform these calculations separately for each functional category and each SIDD level, including the level where there is no destabilization in the upstream regions. The results are displayed on a grid as values of *z_jk_* for each *j* and *k*. This is done for both the GenProtEC and COG functional categorizations, and for the three types of upstream regions described above.

### Sequences analyzed.

The accession numbers of all genomic sequences analyzed in this project are presented in the Dataset S3. The SIDD profiles of all analyzed genomes are accessible through our Web site at http://www.genomecenter.ucdavis.edu/benham. Profiles of complete genomes can be made available on request.

## Supporting Information

Dataset S1 Distribution of SIDD Sites among the GenProtEC Categories (165 KB XLS)

Dataset S2 Distribution of SIDD Sites among COG Categories (72 KB XLS)

Dataset S3 Distribution of Strong SIDD Sites in Transcriptional Regulator Genes in Free-Living Bacteria and Endosymbionts/Parasitic Bacteria (26 KB XLS)

Dataset S4 SIDD Sites in the “Supercoiling” Genes from Microarray Experiments (62 KB XLS)

### Accession Numbers

The Swiss-Prot (http://expasy.org/sprot) accession numbers for the genes discussed in this paper are *ilvG* (P00892), *rpoS* (P13445), *rpoD* (P00579), *gyrA* (P09097), *topA* (P06612), *hupA* (P02342), *himA* (P06984), *crp* (P03020), *lacI* (P03023), *proV* (P14175), *tyrP* (P18199), *ompC* (P06996), *fadL* (P10384), *ilvY* (P05827), *ilvC* (P00510), *cysB* (P06613), *narX* (P10956), *narL* (P10957), *narP* (P31802), *narQ* (P27896), *fnr* (P03019), *ArcA* (P03026), and *ArcB* (P22763).

## Abbreviations

COG: clusters of orthologous groups
IHF: integration host factor
ORF: open reading frame
SIDD: stress-induced DNA duplex destabilization
